# Cervical Abnormalities in Severe Spinal Deformity: A 10‐year MRI Review

**DOI:** 10.1111/os.12673

**Published:** 2020-04-29

**Authors:** Ying Zhang, Ying‐song Wang, Jing‐ming Xie, Zhi Zhao, Tao Li, Ni Bi, Zhi‐yue Shi, Liu‐yuan Chen, Wen‐hua Li, Huai‐li Deng, Yu Lu

**Affiliations:** ^1^ Department of Orthopaedics The 2nd Affiliated Hospital of Kunming Medical University Kunming China

## Abstract

**Objective:**

To investigate the incidence of cervical anomalies (CA), including cervical intraspinal neural axis abnormalities (CIINAA) and/or cervical osseous abnormalities (COA), and the clinical relevance in severe spinal deformities (SSD) at a single center.

**Methods:**

A retrospective study of SSD admitted for spinal surgery from January 2003 to January 2015 was conducted at a single center. Inclusion criteria: patients who present with coronal Cobb over 90° (and/or sagittal cobb ≥90°); and patients with complete imaging and clinical data preoperatively. Exclusion criteria: ankylosing spondylitis, adult onset scoliosis, scoliosis secondary to bone destruction. There were 108 SSD patients who fulfilled the criteria in this research (41 males and 67 females). The mean age of the patients was 18.1 ± 2.7 years (range, 10–45 years). The clinical and radiological data of these patients were reviewed to identify CA and to analyze the relationship between clinical and radiographic characteristics in the population of SSD.

**Results:**

The major curves of scoliosis and segmental kyphosis were 109.1° ± 24.7° and 91.2° ± 29.1°. Cervical abnormalities were detected in 56 patients (51.85%) with 9 different CA, including 28 patients (25.9%) with 6 different COA, 21 patients (19.4%) with 3 different CIINAA, and 7 patients (6.5%) with a combination of COA and cervical intraspinal neural axis abnormalities (CINAA). Basilar invagination and Klippel–Feil syndrome were the most frequent COA. Syringomyelia was the most frequent CINAA. SSD with COA in upper vertebral levels (UVL) had a higher incidence of CINAA than those in subaxial vertebral levels (SVL) (*P* = 0.024) and SSD with multiple COA (mCOA) in UVL had a higher incidence of CINAA than those with single COA (sCOA) (*P* = 0.029). In the present study, 83.9% of the SSD with CA were asymptomatic.

**Conclusion:**

The incidence of CA in SSD was 51.85%, with most presenting with intact neurologic status. As the diversity of COA increased, we found a higher incidence of CINAA, especially in UVL.

## Introduction

The vertebral column is closely related to the spinal cord from an anatomical and embryological perspective. Osseous abnormalities (OA) of the vertebral column associated with spinal deformity are often accompanied by intraspinal neural axis abnormalities (INAA). Previous studies demonstrated that the average prevalence of INAA in patients with idiopathic scoliosis (IS), congenital scoliosis (CS), and severe spinal deformity (SSD) were 17.7%, 38%, and 42.6%, respectively^1^, ^2^, ^3^, ^4^, ^5^, ^6^. Although some published studies have also described OA in CS, the exact prevalence of OA in the population is unknown because most of these patients have presented with intact neurologic status without imaging examination^3^. In contrast, OA can lead to an increased risk of secondary segmental instability and/or spinal cord impingement, which may, in turn, result in neurological dysfunction. Therefore, a higher risk of iatrogenic neurological impairment has been reported during surgery for scoliosis associated with INAA and/or OA than those without^4^, ^7^.

Severe spinal deformity is an uncommon condition, with patients presenting with rigid and severe curve magnitude with acute clinical symptoms, which can require surgical management. In contrast to the more common IS, the treatment for SSD has been identified with more challenges^4^. The mechanisms responsible for the development of SSD and neurological deficit in these disorders, such as higher tension or ischemia of the spinal cord, are postulated to be unique for each^5^. Previous study of SSD indicated that the incidence of INAA in SSD was higher than that in the IS population, and most of these patients presented with intact neurologic status^6^. In these situations, INAA may be a risk factor for neurologic complications during correction of SSD^7^. As a result, all of these conditions potentially place the SSD patients at a much higher risk of developing iatrogenic neurological complications during corrective surgery than other kinds of spinal deformity^6^, ^7^.

The anatomical development and biomechanics of the cervical spine are different from the rest of the spine. Abnormalities are more likely to occur in the cervical spine, either as part of some genetic syndromes or as part of spinal deformity. With the development of imaging techniques, INAA (such as syringomyelia‐S, Chiari malformations‐CM, and Chiari malformation with syringomyelia‐C + S) and/or OA (such as basilar invagination‐BI, atlantoaxial instability‐AI, dysmorphic atlas‐DA, congenital vertebral hypoplasia‐CVH, vertebral facet joint fusion‐VF, and Klippel‐Feil syndrome [KFS]) in the cervical spine, are increasingly being found in patients with scoliosis. According to previous studies, the presence of cervical abnormalities (CA) in any part of the cervical spine often heralds scoliosis, congenital malformations in other organ systems, and various syndromes^1^, ^2^, ^3^, ^4^, ^5^, ^6^. This factor is of most importance to the surgeon to prevent iatrogenic morbidity, whose main attention may be directed toward the patient's deformity. Therefore, the CA often needs to be addressed first to be able to treat the SSD more effectively and/or safely. However, there is little study on CA and the relationship between clinical and radiographic characteristics in the population with SSD.

Therefore, the current study analyzed the imaging data at a single institution treating patients with SSD in the past 10 years in an attempt to: (i) demonstrate the prevalence of CA; (ii) determine the relationship between cervical OA (COA) and cervical intraspinal neural axis abnormalities (CINAA); and (iii) determine clinical and radiographic characteristics correlating with CA.

## Materials and Methods

Institutional review board approval was obtained before this retrospective study. All patients underwent a full clinical examination, plain radiograph of the entire spine, CT, and neural axis MRI of the whole spine. Inclusion criteria for the study were: (i) patients who presented with coronal Cobb over 90° (and/or sagittal Cobb ≥90°) admitted for spinal surgery from 2003 to 2015 at a single institution; and (ii) patients with complete preoperative imaging and clinical data. The orthopaedic and radiological databases, including MRI, etiology and clinical examination, were reviewed to identify CA. The presence or absence of COA and/or CINAA was determined by two orthopaedic surgeons using a double‐blind method independently. Exclusion criteria were: patients with ankylosing spondylitis, adult‐onset scoliosis, scoliosis secondary to bone destruction due to infection, tuberculosis, tumor, trauma, or previous operation.

### *MRI* *Study*

The cervical spine was further stratified into upper vertebral levels (UVL) (C_0_–C_2_), and subaxial vertebral levels (SVL) (C_3_–C_7_). The diversity of COA was also further stratified into single COA (sCOA) (1 kind of OA) and multiple COA (mCOA) (≥2 kinds of OA). The COA, which were detected in UVL as well as SVL (UVL + SVL), were also identified as mCOA.

Cervical anomalies (COA and/or CINAA) within cervical region were investigated by MRI. T1W1 and STIR weighted sagittal, and T1W1 coronal and axial images of the cervical spine regions were examined using 1.5‐T magnetic resonance equipment (Siemens, Aera XQ 1.5T, Germany).

### *Etiology Study*

The patients were divided into three broad groups according to the type of scoliosis: congenital scoliosis (CS), presumed idiopathic scoliosis (PIS), and other types of scoliosis. PIS included all the patients who were diagnosed with “idiopathic” scoliosis at first presentation to the outpatient department.

### *Clinical Examination*

On clinical examination, the presence of neurologic symptoms and abnormal neurologic signs, such as sustained hyperactive reflex, unilateral superficial abdominal reflex, muscle atrophy, motor weakness, sensory loss, and bowel and/or bladder function, were investigated. Neurologic examination was performed separately by at least two spine surgeons. If any abnormalities were found, the patients were reexamined by another physician and consensus was reached following the judgment made by the physician and spine surgeons.

### *Outcome Measures/Observation Index*

#### *Cervical Osseous Abnormalities*

**Figure 1:**
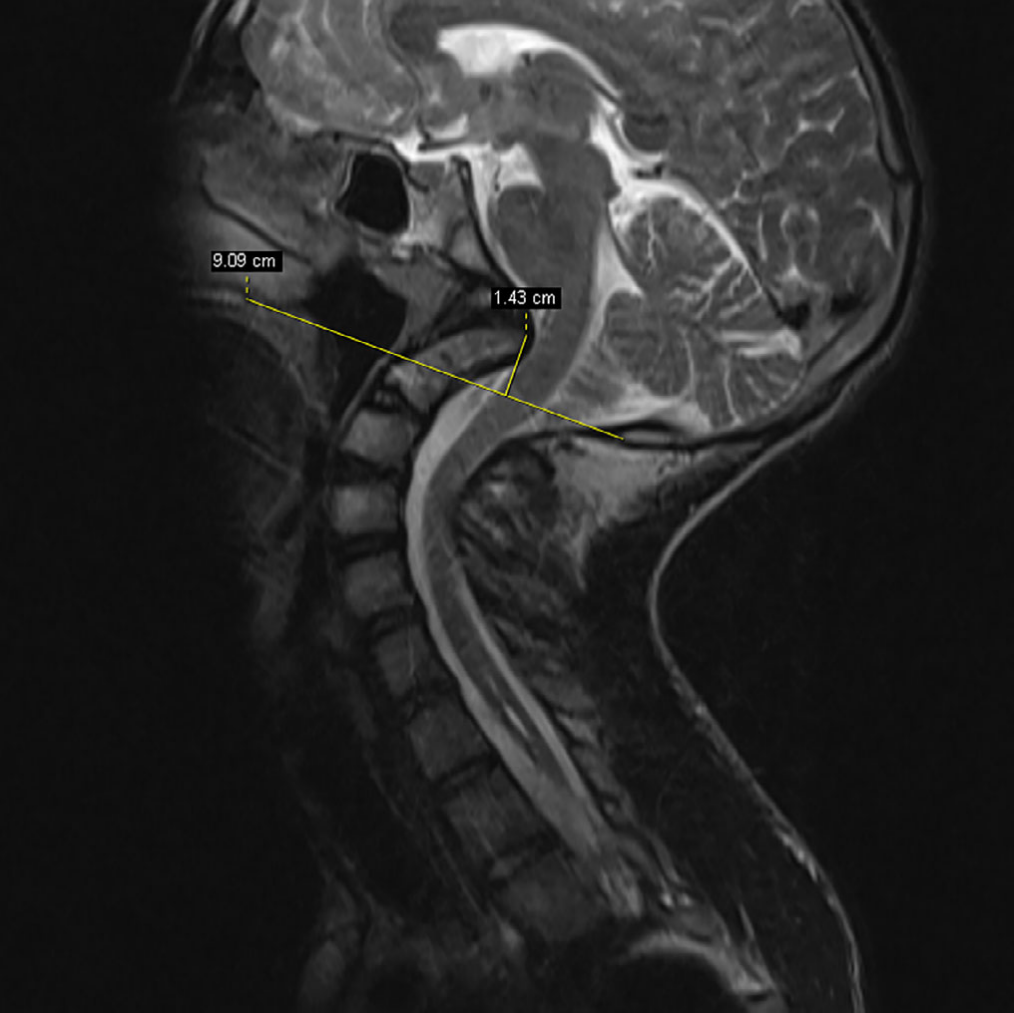
This illustration shows the odontoid process of C_2_ prolapsed into the foramen magnum. In this study, Basilar invagination (BI) was considered if the odontoid tip extended more than 5 mm above the Chamberlain line. The Chamberlain line runs from the hard palate to the opisthion, which is the midpoint of the posterior margin of the foramen magnum.

**Figure 2:**
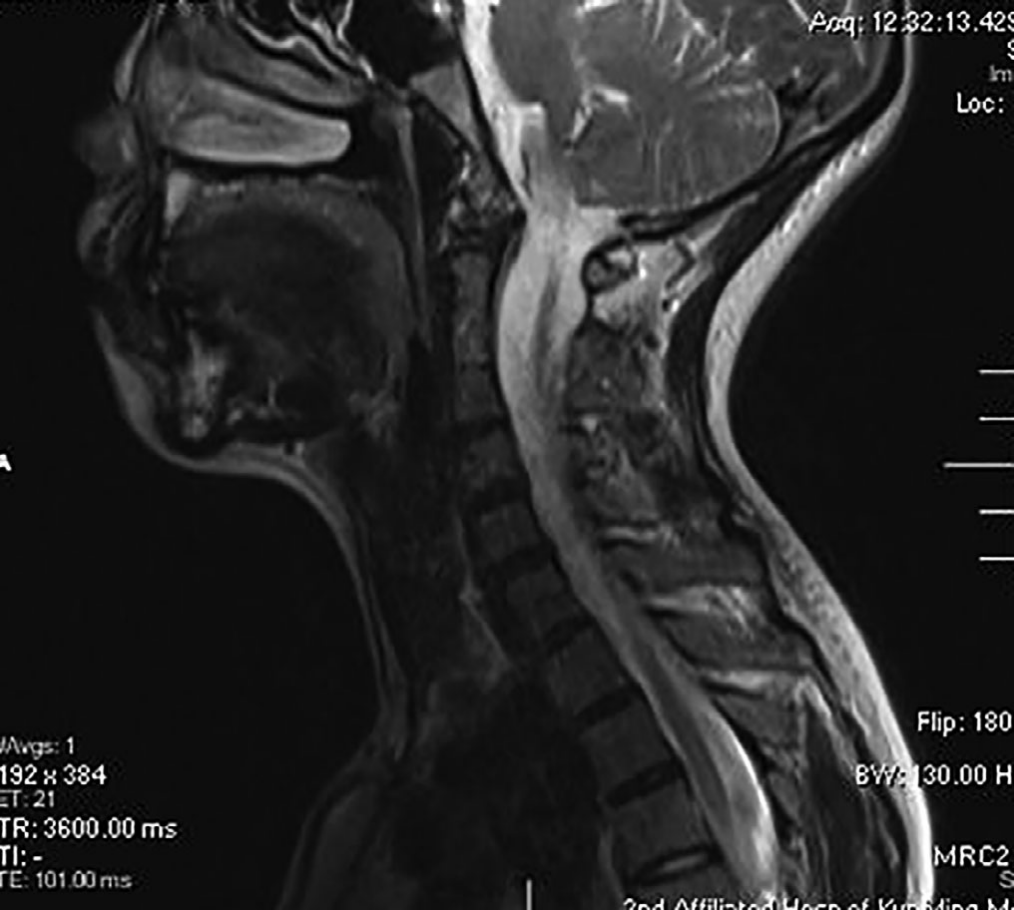
This illustration shows the C_2_–C_4_ fusion. In this study, Klippel–Feil syndrome (KFS) was considered if at least one motion segment of the cervical spine exhibited congenital fusion.

(i) *Basilar Invagination*: Basilar invagination (BI) presents as congenital and/or in acquired forms of the craniovertebral junction COA, where the odontoid process of C_2_ prolapses into the foramen magnum. In this study, BI was considered if the odontoid tip extended more than 5 mm above the Chamberlain line (the Chamberlain line runs from the hard palate to the opisthion, which is the midpoint of the posterior margin of the foramen magnum)^8^ (Fig. 1).

(ii) *Klippel–Feil Syndrome*: Klippel–Feil Syndrome (KFS) is not an uncommon COA and is characterized as improper segmentation or congenital fusion of at least one vertebral motion segment of the cervical spine with or without additional spinal or extraspinal manifestations. In this study, KFC was considered if at least one motion segment of the cervical spine exhibited congenital fusion^9^ (Fig. 2).

#### *Cervical Intraspinal Neural Axis Abnormalities*

**Figure 3:**
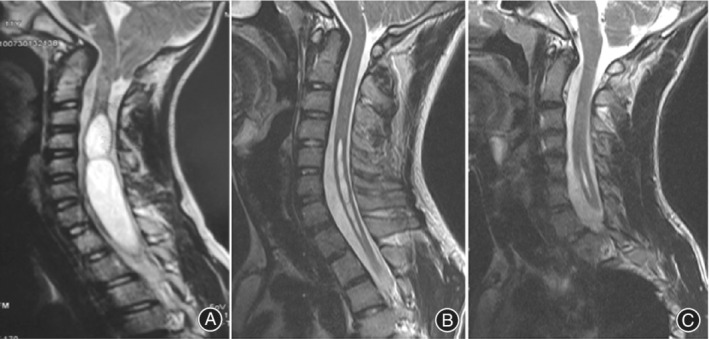
This illustration shows three types of syringomyelia (S): swelling type (A), spindle type (B), and slit type (C). In this study, S was considered if an intramedullary spinal cord cyst or a focal dilatation of the central canal was detected on MRI.

**Figure 4:**
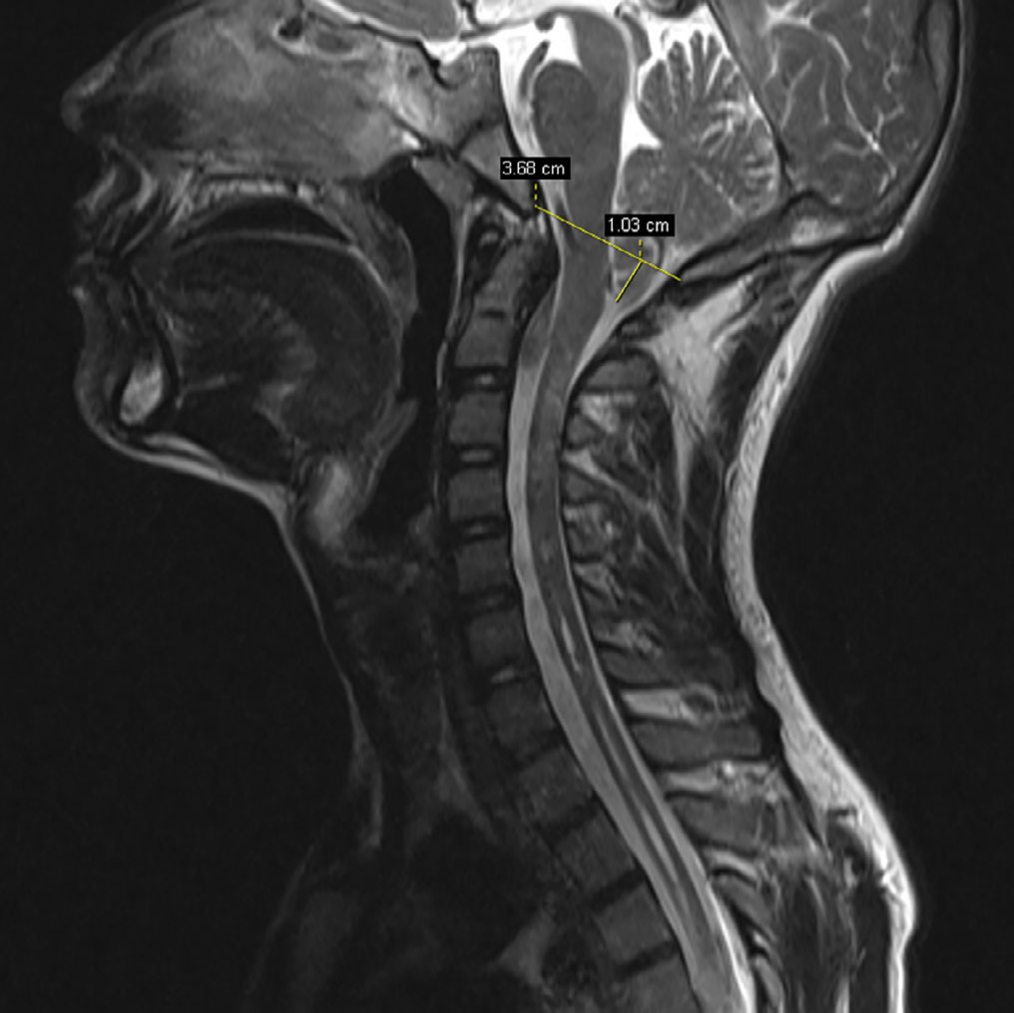
This illustration shows descent of the cerebellar tonsils into the cervical spinal canal. In this study, Chiari malformations (CM) were considered if the tonsils were lying 5 mm or more below the foramen magnum on MRI median sagittal.

(i) *Syringomyelia*: Syringomyelia (S) is CINAA of unknown etiology, which is characterized as either a fluid‐filled, gliosis‐lined cavity within the spinal cord parenchyma or a focal dilatation of the central canal. In this study, S was considered if an intramedullary spinal cord cyst or a focal dilatation of the central canal was detected on MRI, which was hypointense on T1‐weighted imaging and hyperintense on T2‐weighted imaging, without contrast enhancement. Each case of S was classified into one of the three groups: “swelling type,” “spindle type,” or “slit type”^2^(Fig. 3A–C).

(ii) *Chiari Malformations*: Chiari malformations (CM) represent a group of CINAA characterized by descent of the cerebellar tonsils or vermis into the cervical spinal canal. In this study, CM was considered if the tonsils were lying 5 mm or more below the foramen magnum on MRI median sagittal^10^(Fig. 4).

### *Statistical Analysis*

IBM SPSS statistics version 23.0 (IBM, Armonk, NY, USA) was used to analyze all descriptive and comparative data. A χ^2^‐test was administered statistically to analyze the correlations between COA and CINAA. The differences with a *P <* 0.05 were considered statistically significant.

## Results

### *Demographics*

A total of 108 patients who fulfilled the criteria were included. The mean age was 18.1 ± 2.7 years (range, 10–45 yrs.). There were 67 female and 41 male patients. The major curve of scoliosis was 109.1° ±24.7° preoperatively, and the segmental kyphosis was 91.2° ± 29.1°.

### *Cervical Abnormalities*

Cervical abnormalities were detected in 56 patients (56/108, 51.85%; 21 males and 35 females) and 9 different CA were detected. The total frequency of detected CA was 66 in our study. The most frequent CA included BI (31.8%), KFS (19.7%), and S (15.2%), followed by CM + S (10.6%), VF (7.6%), CVH (6.1%), CM (6.1%), AI (1.5%), and DA (1.5%) (Table 1).

**Table 1:** Frequency of all CA in SSD

	Abnormalities	Frequency	Percentage (%)
CA	BI	21	31.8
	KFS	13	19.7
	VF	5	7.6
	CVH	4	6.1
	AI	1	1.5
	DA	1	1.5
CINAA	S	10	15.2
	CM + S	7	10.6
	CM	4	6.1
Total	9	66	100

AI, atlantoaxial instability; BI, basilar invagination; CM + S, Chiari malformation combined with syringomyelia; CA, cervical anomalies; CINAA, cervical intraspinal neural axis abnormalities; CM, Chiari malformation; COA, cervical osseous abnormalities; CVH, congenital vertebral hypoplasia; DA, dysmorphic atlas; KFS, Klippel–Feil syndrome; S, syringomyelia; SSD, severe spinal deformity; VF, vertebral facet joint fusion.

### *Cervical Osseous Abnormalities*

**Figure 5:**
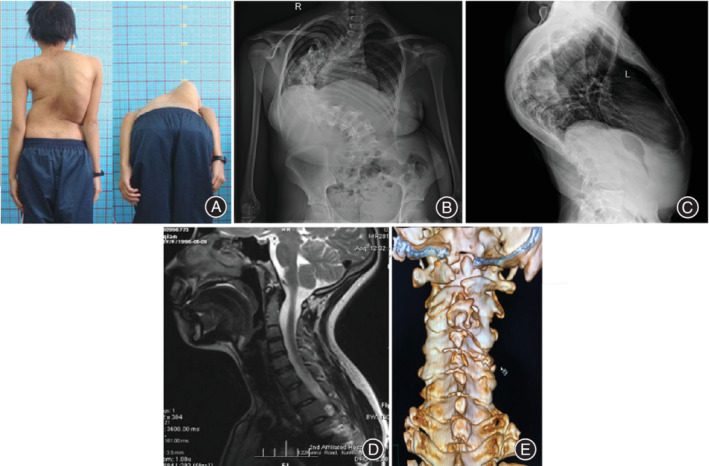
A 20‐year‐old female with severe kyphoscoliosis associated with cervical osseous abnormalities (COA). The preoperative appearance reveals spinal deformity (A). The preoperative AP and lateral radiographs show the severe kyphoscoliosis (B, C). The MRI and CT scanning of the cervical spine (D, E) show a variety of COA manifested in combination.

Cervical osseous abnormalities were detected in 28 patients (28/108, 25.9%; 13 males and 15 females) and 6 different COA were detected. The total frequency of detected COA was 45 (45/66, 68.2%) in our study. Of these, 10 (10/28, 35.7%) patients with frequency of 23 (23/45, 51.1%) were detected with COA in UVL. BI was detected in 8 patients (8/10, 80.0%), which was the most common COA in UVL, followed by DA (1/10, 10%), and AI (1/10, 10%). Overall, 13 (13/28, 46.4%) patients with frequency of 22 (22/45, 48.9%) were detected with COA in SVL. KFS was detected in 4 patients (4/13, 30.8%), which was the most common COA in SVL (Tables 1, 2) (Fig. 5).

**Table 2:** Description of cervical abnormalities and preoperative neurological deficits in SSD

	COA			CIINAA			COA + CIINAA
Variable	UVL	SVL	UVL + SVL	CM	S	CM + S	
Number of cases	10	13	5	4	10	7	7
PrND	3	0	1	0	2	1	2
Etiologies							
PIS	7	6	3	4	8	6	6
CS	2	7	2	0	2	0	0
Others	1	0	0	0	0	1	1

CINAA, cervical intraspinal neural axis abnormalities; COA, cervical osseous abnormalities; CM, Chiari malformation; S, syringomyelia; CM + S, Chiari malformation combined with syringomyelia; CS, congenital scoliosis; PIS, presumed idiopathic scoliosis; PrND, Preoperative neurological deficits; SSD, severe spinal deformity; SVL, subaxial vertebral levels (C_3_–C_7_); UVL, upper vertebral levels (C_0_–C_2_).

### *Cervical Intraspinal Neural Axis Abnormalities*

**Figure 6:**
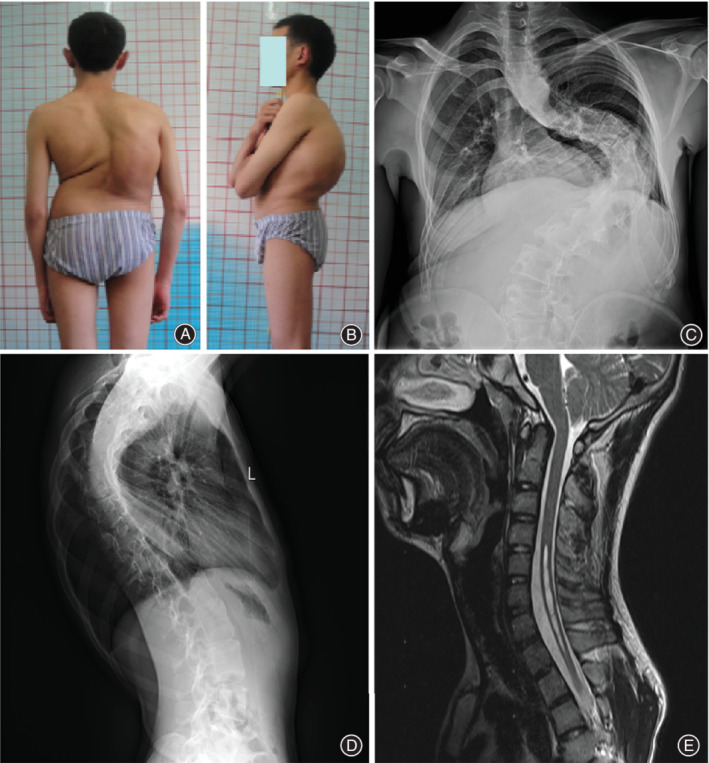
An 18‐year‐old boy with severe kyphoscoliosis associated with cervical intraspinal neural axis abnormalities (CIINAA). The preoperative appearance reveals spinal deformity (A, B). The preoperative AP and lateral radiographs show the severe kyphoscoliosis with trunk imbalance (C, D). The MRI shows the Chiari malformations and syrinx in the cervical region (E).

Cervical intraspinal neural axis abnormalities were detected in 21 patients (21/108, 19.4%; 5 males and 16 females) and 3 different COA were detected. The total frequency of detected CINAA was 21 (21/66, 31.8%) in our study. The most common CINAA was S, which was detected in 10 patients (10/21, 47.6%), followed by C + S (7/21, 33.3%) and CM (4/21 19.0%). For the S, 1/10 (10.0%), 3/10 (30.0%), and 6/10 (60.0%) were swelling, spindle, and slit types, respectively (Tables 1, 2) (Fig. 6).

### *Cervical Osseous Abnormalities combined with Cervical Intraspinal Neural Axis Abnormalities*

**Figure 7:**
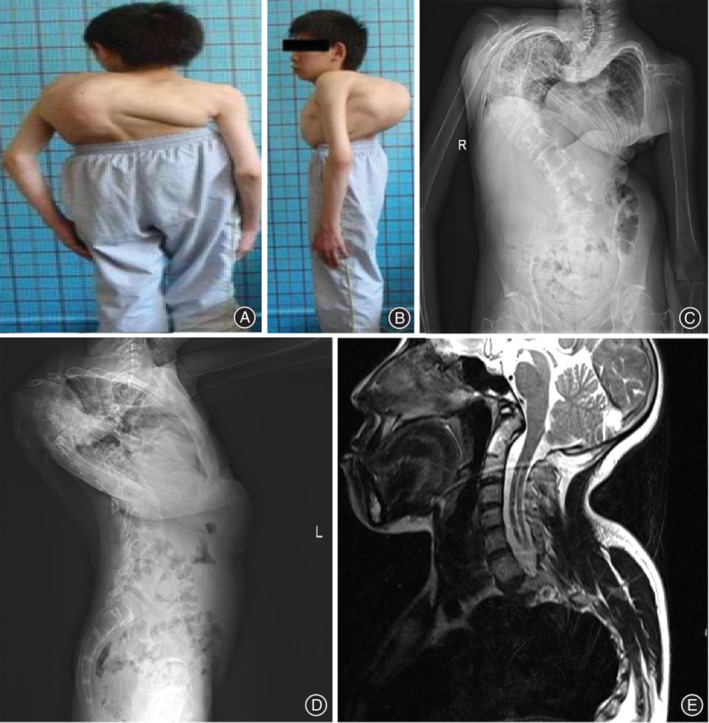
A 17‐year‐old boy with severe kyphoscoliosis associated with cervical osseous abnormalities (COA) and cervical intraspinal neural axis abnormalities (CIINAA). The preoperative appearance reveals spinal deformity (A, B). The preoperative AP and lateral radiographs show the severe kyphoscoliosis (C, D). The sagittal MRI scanning of the cervical spine shows the C_2_–C_3_ fusion, basilar invagination, and Chiari malformation with expanding central canal (E).

Cervical osseous abnormalities combined with CINAA were detected in 7 patients (7/108, 6.5%; 3 males and 4 females) in our study (Table 2)(Fig. 7).

### *Correlations between Cervical Osseous Abnormalities and Cervical Intraspinal Neural Axis Abnormalities*

There were 7 patients who were detected with a combination of COA and CINAA. As to the vertebral levels, 6 of 16 (37.50%) patients with COA in UVL were detected with CINAA, 1 of 5 (20.00%) patients with COA in UVL + SVL were detected with CINAA, and none of 13 patients with COA in SVL were detected with CINAA. SSD patients with COA in UVL had a higher incidence of CINAA than those in SVL (*P* = 0.024). As to the diversity of COA in UVL, 4 of 4 (100.00%) patients with mCOA were detected with CINAA and 3 of 14 (21.42%) patients with sCOA were detected with CINAA. SSD patients with mCOA in UVL had a higher incidence of CINAA than those with sCOA (*P* = 0.029) (Tables 3, 4).

**Table 3:** Correlations between COA and CINAA in different vertebral levels

Vertebral levels of COA	CINAA		*P* **‐**value
	Yes	No	
UVL SVL	6 0	10 13	0.024
UVL UVL + SVL	6 1	10 4	1
SVL UVL + SVL	0 1	13 4	0.278

CINAA, cervical intraspinal neural axis abnormalities; COA, cervical osseous abnormalities; SVL, subaxial vertebral levels (C_3_–C_7_); UVL, upper vertebral levels (C_0_–C_2_).

**Table 4:** Correlations between COA and CINAA according to diversity of COA in UVL

Diversity of COA	CINAA		*P‐*value
	Yes	No	
sCOA	3	11	0.029
mCOA	4	0	

CINAA, cervical intraspinal neural axis abnormalities; COA, cervical osseous abnormalities; UVL, upper vertebral levels.

### *Etiology*

There were 82 PIS patients (82/108, 75.9%), 22 CS patients (22/108, 20.4%), and 4 other patients (4/108, 3.7%). As to the SSD patients with COA, there were 16 PIS patients (16/28, 57.1%), 11 CS patients (11/28, 39.3%), and 1 other patient (1/28, 3.6%). As to the SSD patients with CINAA, there were 18 PIS patients (18/21, 85.7%), 2 CS patients (2/21, 9.5%), and 1 other patient (1/21, 4.8%). As to the SSD patients with COA combined with CINAA, there were 6 PIS patients (6/7, 85.7%) and there was 1 other patient (1/7, 14.3%) (Table 2).

### *Physical Findings*

On clinical examination, 17 of 108 patients (17/108, 15.7%) had abnormal neurologic signs, 9 of which (9/17, 52.9%) had CA, including 4 with COA, 3 with CINAA, and 2 with COA combined with CINAA. Most of the SSD patients with CA (47/56, 83.9%) were asymptomatic (Table 2).

## Discussion

Previous studies demonstrated that a considerable proportion of scoliosis was associated with INAA and OA, with most in the cervical region, especially in SSD patients^1^, ^2^, ^3^, ^5^, ^6^. High risk of neurological complications has been reported during corrective surgery of scoliosis associated with INAA and is one of the independent risk factors of neurologic deficits in the treatment of SSD^4^, ^7^, ^11^. These situations place SSD patients at risk for iatrogenic neurologic injury. To prevent potential neurological complications, CA need to be addressed before the treatment of SSD. Documenting the incidence, varieties of CA in SSD patients will help surgeons to choose a proper treatment.

### *The Incidence of Cervical Abnormalities in Severe Spinal Deformities*

All the patients in this study were SSD patients, with Cobb angle of the main curve greater than 90° in the coronal plane and/or sagittal plane. We report a higher incidence of CA (51.85%) than other studies which demonstrated that the average prevalence of CA associated with mild‐or‐moderate scoliosis was approximately 15%–40%^1^, ^2^, ^3^, ^12^. As to the etiology, most patients in this study were PIS patients (82/108, 75.9%). Although, the exact relationship between CA and scoliosis remains to be defined, IS is an exclusion diagnosis; that is, other causes of deformity must have been ruled out by physical examination or imaging. Therefore, one of the possible reasons for this result was that some of these patients with CA were not screened by medical examination and/or were diagnosed by MRI at early onset until final severe curve magnitude. Furthermore, genetic syndromes are associated with varied patterns of CA involvement. It is important to be vigilant in the diagnosis of CA in patients with scoliosis who may have associated genetic syndromes. Due to lack of awareness and difficult diagnosis procedures for genetic syndromes, some of these patients were also categorized as PIS at early onset.

### *Cervical Osseous Abnormalities in Severe Spinal Deformities*

The mechanism responsible for the development of COA has been variously discussed by researchers^12^, ^13^, ^14^, ^15^, ^16^, ^17^, ^18^. However, the exact prevalence of COA is unknown, as most individuals who have COA are asymptomatic. The ossification centers of UVL (C_0_–C_2_) may be an insufficient amount of paraxial mesoderm, leading to a wide variety of congenital, hereditary OA existence in this region, either individually or in combination. Once the stage is set by a congenital COA of this sort, further developmental and acquired phenomena may supervene, such as BI. Commonly reported abnormalities in this region include CVH, BI, AI, DA, VF, occipitalization of the atlas, a dysmorphic atlas, and a dysmorphic dens^15^, ^16^. Similarly, in examining 108 SSD patients, we found a higher incidence of COA (28/108, 25.9% patients). Among those with COA, 10 (10/28, 35.7%) were detected with a variety of COA manifested separately or in combination in UVL with a frequency of 23 (23/45, 51.1%), including VF, CVH, AI, and DA. Higher incidence of congenital and/or hereditary COA in UVL resulted in higher incidence of developmental and acquired COA–BI, which was the most common COA (8/10, 80.0% patients) in UVL.

Although a wide variety of COA can occur in UVL, COA in SVL is not uncommon. Perhaps the most common congenital malformation of the SVL is KFS, which refers to any congenital fusion of two or more cervical vertebrae, with or without the classic triad^9^, ^13^, ^14^, ^15^, ^16^, ^17^. Numerous musculoskeletal anomalies are associated with KFS, with the most common being scoliosis (usually congenital), which occurs in up to 60% of patients^13^, ^14^, ^15^, ^16^. In addition to KFS, a variety of formation‐segmentation COA can occur in SVL, as they do in other parts of the spine. These include midline vertebral body clefts, sagittal and coronal hemivertebrae, hypoplasia or complete absence of a vertebra, absence of a pedicle, and block vertebrae. In this study, 13 of 28 patients with COA (13/28, 46.4%) were detected in SVL and a high percentage of patients (4/13, 30.8% patients) had been detected with KFS, which was the most common COA in SVL.

### *Cervical Intraspinal Neural Axis Abnormalities in Severe Spinal Deformities*

The vertebral column and spinal cord are closely related from an anatomical and developmental perspective. During the early stage of development, bony elements of the spine form in coordination with the neural tube^12^, ^13^, ^14^. In addition, any disturbance in normal cerebrospinal fluid (CSF) caused by the OA and/or INAA, such as BI, CM, obstructing normal CSF flow from the cranial to spinal compartment during the normal cardiac cycle or increasing arterial pressure that can cause CSF to enter directly into the parenchyma of the cord *via* perivascular spaces, may lead to syringomyelia. In addition, there is currently no doubt that there exists a relationship among CM, S, and scoliosis. Therefore, a significant number of patients with spinal deformity have both INAA and OA, particularly those associated with scoliosis and kyphosis. Some studies have suggested the incidence of INAA associated with OA in CS to be as high as 30%–53%, and the incidence of INAA in cervical region from 56.6% to 70%^5^, ^6^, ^18^, ^19^. With the higher incidence of COA in the present study, 19.4% (21/108) of all SSD patients were detected with CINAA and 6.5% (7/108) of patients were detected with a combination of COA and CINAA. The most common CINAA was S (10/21, 47.6%) and most were slit types (6/10, 60.0%).

### *Relationship Between Cervical Osseous Abnormalities and Cervical Intraspinal Neural Axis Abnormalities in Severe Spinal Deformities*

Another purpose of this study is to determine what the relationship between COA and CINAA is in a population with SSD. Previous studies have traditionally focused on the relationship between complexity of CS and the incidence of concurrent INAA. For example, Basu *et al*. reported that CS patients with mixed malformations of the vertebrae were found to have an incidence of 40% of INAA, while failures of formation and segmentation alone had incidences of 30% and 40%, respectively^20^. Trenga *et al*. also reported that complex bony abnormalities or mixed deformities of segmentation and formation had a higher incidence of INAA in CS patients^18^. In the present study, all 7 patients with a combination of COA and CINAA were detected with COA in UVL (1 in UVL + SVL group, 6 in UVL group). Therefore, SSD patients with COA in UVL had a higher incidence of CINAA than those in SVL *(P* = 0.024). Moreover, as to the diversity of COA, more than half of CINAA were detected in the mCOA group (1 in UVL + SVL group, 3 in mCOA group). Although the sample size was small, our result indicated that patients with mCOA in UVL had a higher incidence of CINAA than those with sCOA (*P* = 0.029). The results of this study suggested there is a possible link between CINAA and vertebral levels and/or diversity of COA in the SSD population.

### *Clinical Relevance of Cervical Abnormalities in Severe Spinal Deformities*

The compression of the nervous system may be by bone, soft tissue, or by indirect compromise of the blood supply. Pressure from an asymmetrically expanding CA may be imparted to the medial nuclear group of cells and irreversible damage by the CA to nerve cells may present with neurologic symptoms and abnormal neurologic signs. Some previous studies suggested that the incidence of MRI abnormalities in IS with a negative history and physical examination was up to approximately 20%^1^, ^2^, ^3^. As to SSD, the incidence of INAA was 42.6% and most of presented with intact neurologic status^6^. In this series, most of the SSD patients with CA (47/56, 83.9%) are asymptomatic. The more frequent CA without clinical manifestations in our research may influence orthopaedic surgical treatment in SSD patients.

### *Limitations*

This study has several limitations. First, it is difficult to launch a prospective protocol in the low incident severe spinal deformities. This retrospective study precluded a powerful statistical analysis, or the ability to draw any strong conclusions. Second, a relatively smaller sample size in a single institute may lead to sample bias. However, the results of this research still increase knowledge for the treatment of SSD. Multiple‐center, large‐sample, clinical trials are required in the future to confirm our conclusion.

### *Conclusion*

Our study therefore provides additional evidence to suggest that the incidence of CA in SSD was 51.85%, with most patients presenting with intact neurologic status. A wide variety of CA in SSD patients may develop as single or multiple abnormalities in the same individual and involve both osseous and neural structures. It is also suggested that patients with SSD are indicated for preoperative routine imaging examination, including the cervical spine, even when patients are asymptomatic and have a normal physical examination.
